# Editorial: Novel Therapeutic Interventions Against Infectious Diseases: COVID-19

**DOI:** 10.3389/fphar.2022.852078

**Published:** 2022-03-14

**Authors:** Sugunadevi Sakkiah, Brijesh Kumar Singh, Keun Woo Lee, Chandrabose Selvaraj

**Affiliations:** ^1^ National Center for Toxicological Research, Jefferson, AR, United States; ^2^ Duke-NUS Medical School, Singapore, Singapore; ^3^ Department of Biochemistry, Gyeongsang National University, Jinju, South Korea; ^4^ Department of Bioinformatics, Alagappa University, Karaikudi, India

**Keywords:** COVID - 19, SARS – CoV – 2, drug repurpose, molecular docking, virtual screeening

The research topic “Novel Therapeutic Interventions Against Infectious Diseases: COVID-19” intends to examine, at the molecular level, the mechanisms of SARS-CoV-2 infection and their potential inhibition through computational or experimental approaches. Drug targets for SARS-CoV-2 infections and macromolecules responsible for the virion's binding to the host receptor protein are described in detail. The 15 research articles in this issue, each focusing on a different aspect of the fight against SARS-CoV-2, use a variety of interdisciplinary approaches, including computational chemistry, biochemical analyses, and biological activity testing. Contributing authors have searched for novel leads from the available natural substances, new chemical entities, and FDA-approved drugs to target SARS-CoV-2. Indari et al. present a comprehensive update on FDA-approved drugs for repurposing, namely chloroquine, hydroxychloroquine, remdesivir, lopinavir-ritonavir, favipiravir, ribavirin, azithromycin, umifenovir, and oseltamivir as well as convalescent plasma therapy used as antiviral therapy against SARS-CoV-2. Preclinical and clinical findings, treatment regimens, pharmacokinetics, and drug–drug interactions are discussed in this review. Some clinically approved medications have been proposed as potential anti-SARS-CoV-2 options as a result of this repurposing strategy. Yadalam et al. performed a computational study to identify the essential oil components as SARS-CoV-2 antivirals, especially in the pre-procedural mouth rinses for dental settings. Pre-procedural mouth rinses are helpful in decreasing viral particles in the oral cavity, since most of COVID-19 dissemination occurs due to the virus' presence in the mouth. Through the molecular docking and conceptual density functional theory (DFT) approach, the antiviral efficacy of essential oil components are studied against the receptor binding domain (RBD) of the spike protein. The compounds cuminal, carvacrol, myrtanol, and pinocarveol were found to be highly active by showing strong interactions with the RBD and shown to be active based on the correlation between the structure and the activity of the compounds. They recommend these components to be included as pre-procedural mouth rinses for dental procedures. Cai et al. has come up with a rehabilitation strategy by applying intermittent hypoxic preconditioning (IHP) and also showed how IHP can be a beneficial treatment strategy in the management of COVID-19. IHP, a non-drug alternative therapy for COVID-19 management, showed beneficial effects related to the impact of oxidative stress, inflammation, and the immune response. Li and Peng have reported the strategy and challenges and described recent progress in identifying broad-spectrum antivirals through drug repurposing, by classifying them into direct-acting repurposed antivirals (DARA) and host-targeting repurposed antivirals (HTRA). In addition, they have summarized and examined the putative mechanisms of action of repurposed antivirals with potential broad-spectrum effectiveness against a range of viruses. Xiao et al. reports the potential of myricetin for the inhibition of the main protease in SARS-CoV-2, with a 3 μM IC_50_ in the enzyme assay. They also reported myricetin as having potent effect on bleomycin-induced pulmonary inflammation, by inhibiting the infiltration of inflammatory cells and the secretion of inflammatory cytokines IL-6, IL-1α, TNF-α, and IFN-γ. Rattis et al. have reviewed the therapeutic potential of curcumin, which interferes at different time points during the infection caused by SARS-CoV-2. This review has strategically contributed to the relentless search for therapies that can act on the combat of COVID-19, in addition to providing targets for future studies using the curcumin as an adjuvant treatment to COVID-19. Wang et al. stated the importance of pulmonary surfactants (PS) in treating acute respiratory distress syndrome in COVID-19. The lack of efficacy reported so far is attributed to the insufficient delivery of PS to the lungs; thus, research has been initiated to investigate new drug delivery systems for improving the PS delivery directly to the lungs. In support to that, they have integrated the data on PS with reference to pulmonary physiology and infection with its possible therapeutic benefit in COVID-19 patients. Hsu et al. have found the potency of remdesivir and cyclosporine that synergistically inhibit the human coronaviruses OC43 (HCoV-OC43) and SARS-CoV-2 by showing inhibitory activity against HCoV-OC43 in HCT-8 and MRC-5 cells. This study, suggests that the combination of remdesivir and cyclosporine merits further study as a possible treatment for COVID-19 complicated by a cytokine storm. Du et al. elucidate the add-on effect of honeysuckle for the treatment of COVID-19 with a meta-analysis. Honeysuckle combined with conventional therapy may be beneficial for the treatment of COVID-19 in improving lung CT, clinical cure rate, clinical symptoms, and laboratory indicators and reducing the rate of conversion to severe cases. Wang et al. highlight the role of high-density lipoprotein (HDL) in COVID-19 by analyzing the pathophysiological characteristics of COVID-19, the pleiotropic properties of HDL, the changes and clinical significance of HDL, and prospect of HDL-targeting therapy. They also suggest that the HDL level-raising pharmacological compounds, such as cholesteryl ester transfer protein (CETP) inhibitors and fibrates, which are already in the preclinical research stage, may be considered as potential treatments for patients with COVID-19. Basit et al. have designed the short peptides and report those peptides for blocking interactions between SARS-COV-2 and human ACE2. The RBD is highly conserved and is also a potential target for blocking its interaction with human cell surface receptor. For this, they have chosen the amino acid regions 21–40 and 65–75 of ACE2 as scaffold for the *de novo* peptide design, and those peptides are potentially strong candidates for the blocking of protein–protein interactions. Kulkarni et al. 2021 have characterized the phytocompounds from the *Ulva intestinalis* L. and report its action against the SARS-CoV-2 spike glycoprotein RBD. Some compounds, namely, 2,4-di-tert-butylphenol (2,4-DtBP); doconexent; 4,8,13-duvatriene-1,3-diol (DTD); retinoyl-β-glucuronide 6′,3′-lactone (RBGUL); and retinal had showed better binding affinity for RBD. Similarly, Kumar et al. have also identified the phytocompounds from sesame against SARS-CoV-2 main protease drug target through molecular docking and dynamics. They have identified four natural metabolites from sesame, namely, sesamin, sesaminol, sesamolin, and sesamolinol through docking and dynamics approach with the Mpro and reported their interactions insights. Rizvi et al. have shown the effect of prophylactic use of intranasal oil formulations in the hamster model of COVID-19. They have reported the prophylactic application of two intranasal formulations provided by the National Medicinal Plant Board (NMPB), anu oil and til tailya, in the hamster model of SARS-CoV-2 infection. Their molecular analysis using mRNA expression profiling indicated the reduced expression of pro-inflammatory cytokine genes, including Th1 and Th17 cytokines for both the intranasal formulations as a result of decreased viral load. Fred et al. have performed *in vitro* studies on antidepressant and antipsychotic drugs that reduce the viral infection by SARS-CoV-2. Their results show that approved drugs of antidepressants, including fluoxetine, citalopram, reboxetine, and imipramine, as well as antipsychotic compounds chlorpromazine, flupenthixol, and pimozide inhibited the infection by pseudotyped viruses with minimal effects on cell viability. Overall, the “Novel Therapeutic Interventions Against Infectious Diseases: COVID-19” research topic gives an updated summary of molecular and mechanical insights towards identifying COVID-19 therapeutic targets for the identification or repurposing of molecules to be used against the virus.

